# Gastric Carcinoid with Hypergastrinemia: Report of Three Cases

**DOI:** 10.1155/2010/348761

**Published:** 2010-09-22

**Authors:** Katsuyoshi Furumoto, Hidenobu Kojima, Masayuki Okuno, Hiroaki Fuji, Rei Mizuno, Tomohiko Mori, Daisuke Ito, Masafumi Kogire

**Affiliations:** Department of Surgery, Kishiwada City Hospital, 1001 Gakuhara-cho, Kishiwada-shi, Osaka 596-8501, Japan

## Abstract

We report 3 cases of gastric carcinoids with hypergastrinemia. *Case 1*: A 60-year-old man had a 2 cm carcinoid of the stomach and underwent partial resection. Involvement of the muscularis propria and lymph nodes metastasis were observed microscopically. Follow-up gastroscopy revealed another carcinoid lesion and total gastrectomy was performed. *Case 2*: A 67-year-old woman with multiple carcinoids of the entire stomach underwent antrectomy. No growth of residual tumors has been detected so far. *Case 3*: A 61-year-old man had a tumor near the esophagogastric junction and underwent total gastrectomy. Carcinoid component was diffusely intermingled with adenocarcinoma in the tumor and invaded into the subserosa. In all 3 cases, the serum gastrin level was high and atrophic gastritis was microscopically observed. Carcinoid tumor in Case 3 was different from those in Cases 1 and 2 and interestingly, gastric carcinoid with hypergastrinemia showed various types of appearance.

## 1. Introduction

Gastric carcinoids originate from the foregut and are derived from enterochromaffin-like (ECL) cells, which are the main neuroendocrine cells in the gastric mucosa. Gastrin acts directly on ECL cells to induce hyperplasia, dysplasia, and, eventually, neoplasia or carcinoid [1]. In patients with chronic atrophic gastritis, decreased gastric acid secretion stimulates the secretion of gastrin, and ECL cells transform into a carcinoid. In this paper, we present 3 cases of gastric carcinoids in atrophic gastritis with hypergastrinemia. One patient had a relatively large tumor with lymph node metastasis, another patient had multifocal small tumors, and the third patient had a large tumor mixed with poorly differentiated adenocarcinoma. A gastric carcinoid with hypergastrinemia is usually small, multiple, and located within the mucosa or submucosa. We experienced 3 different types of gastric carcinoids.

## 2. Case Presentation

**Case 1**

**Figure 1:**
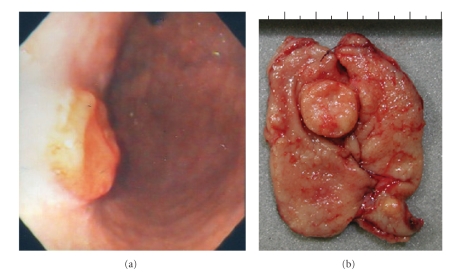
(a) Gastroscopy showed a round tumor at the anterior wall of the gastric body. (b) The resected tumor was 2 cm in diameter.

**Figure 2:**
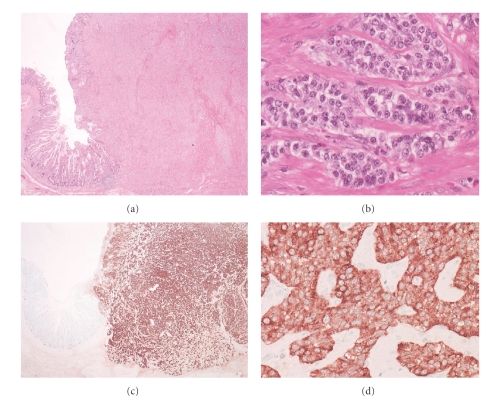
(a) Histological examination of the partially resected tumor revealed carcinoid with muscularis propria involvement (H&E ×4). (b) Carcinoid was consisted of uniform cells with round-shaped nuclei with ribbon-like structure (H&E ×40). (c), (d) The tumor cells showed immunoreactivity for synaptophysin (synaptophysin, (c) ×4, (d) ×40).

**Figure 3:**
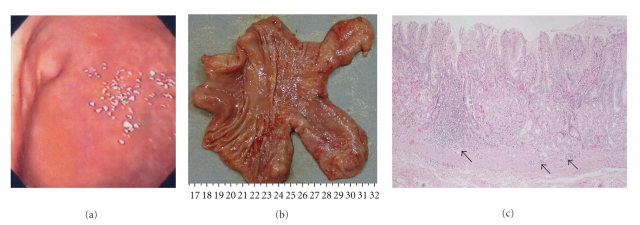
(a) Follow-up gastroscopy revealed another polypoid lesion in the residual stomach. (b) Resected whole stomach. (c) Numerous endocrine cell micronests (arrows) were observed widely in the muscularis mucosae of the stomach (H&E, ×4).

A 60-year-old man, who suffered from cerebral palsy due to measles encephalopathy at the age of 6 months, became unable to eat and underwent gastroscopy. A round tumor was detected at the anterior wall of the middle gastric body, and biopsy revealed a carcinoid (Figure 1(a)). No other polypoid lesions were detected. The level of serum gastrin was very high (4100 pg/mL (normal range: <200 pg/mL)) and the antiparietal cell antibody test was positive. No gastrin-producing tumor was detected by radiologic examination. Because of his poor activity of daily life and benign nature of the carcinoid, his family did not agree any surgical treatment, and endoscopic resection was first considered; however the tumor was too large to be removed endoscopically. Due to the persistent symptom, they accepted only a reduced surgery, and partial resection of the stomach was performed and metastatic lymph nodes were dissected. The tumor was 2 cm in diameter (Figure 1(b)) with involvement of the muscularis propria (Figure 2(a)). These uniform cells (Figure 2(b)) were strongly positive for chromogranin A and synaptophysin (Figures 2(c) and 2(d)). High lymphatic permeation was observed. Seven months later, follow-up gastroscopy revealed another polypoid lesion in the residual stomach (Figure 3(a)). The biopsy revealed a carcinoid and hypergastrinemia was persistent. Total gastrectomy was performed (Figure 3(b)), and microscopic evaluation showed numerous endocrine cell micronests (ECMs; size: 0.1~1.75 mm), which were diffusely observed in the stomach (Figure 3(c)). Regional lymph node metastasis was observed again. The gastric mucosa was diagnosed as compatible with type A atrophic gastritis. Mucosal atrophy was evident in the fundus but not in the antrum. The level of serum gastrin became within normal range.

**Case 2**

**Figure 4:**
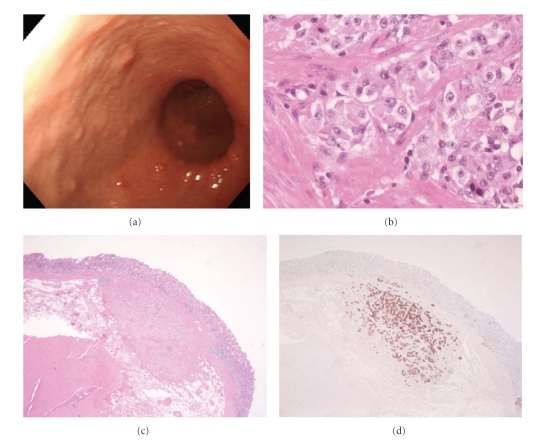
(a) Gastroscopy revealed multiple elevated lesions at the whole stomach. (b) Pathological analysis of the tumors after antrectomy showed carcinoid consisted of uniform cells with round-shaped nuclei with rosette-like or nodular solid structure (H&E ×40), (c) with submucosal invasion (H&E ×4). (d) The tumor cells were positive for chromogranin A staining (chromogranin A ×4).

**Figure 5:**
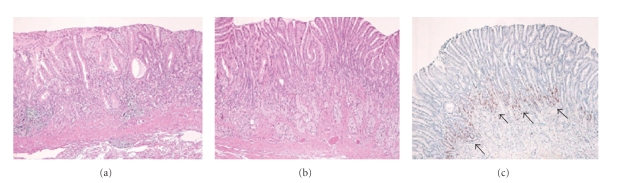
Background gastric mucosa of the resected stomach was type A gastritis microscopically. (a) Atrophy of gastric glands in the mucosa of gastric body was shown (H&E ×4). (b) In the mucosa of antrum, no apparent atrophy of the pyloric glands was observed (H&E ×4). (c) Hyperplasia of gastrin-producing cells was detected (arrows) (gastrin staining ×4).

A 67-year-old woman experienced epigastric pain and nausea for the past 2 months. Gastroscopy revealed lots of elevated lesions (all were less than 5 mm in diameter) in the whole stomach (Figure 4(a)), and the biopsy showed a carcinoid. She was recommended to undergo total gastrectomy and was referred to our department. On the basis of a very high level of serum gastrin (>3000 pg/mL), positive antiparietal cell antibody test, and atrophic gastritis (which was observed macroscopically and microscopically), we diagnosed her condition as typical type 1 carcinoid and performed antrectomy. Pathological analysis showed that the resected tissue was a carcinoid (Figure 4(b)) with invasion into the submucosa (Figure 4(c)). The tumor cells were positive for chromogranin A (Figure 4(d)) and synaptophysin. Type A gastritis was observed under the background of gastric mucosa of the antrectomy specimen, which was characterized by atrophic gastric glands of gastric body and decreased number of parietal cells (Figure 5(a)). In the mucosa of antrum, no apparent atrophy of the pyloric glands (Figure 5(b)) and hyperplasia of gastrin-producing cells were detected (Figure 5(c)). After surgery, serum gastrin level immediately decreased to the normal range. More than 12 months have passed since antrectomy, and no apparent growth of the residual tumors has been detected by gastroscopy so far.

**Case 3**

**Figure 6:**
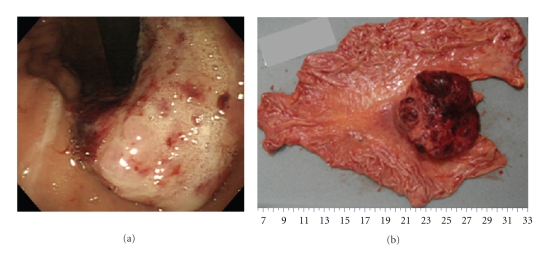
(a) Gastrcosopy revealed a large gastric tumor near the esophagogastric junction. (b) Total gastrectomy was performed, and the resected tumor was approximately 8 cm in diameter.

**Figure 7:**
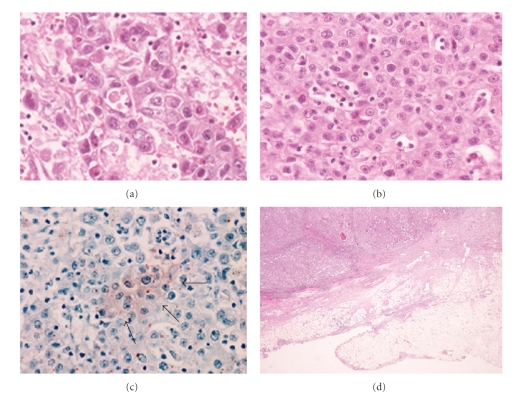
(a), (b) Microscopic findings of the resected specimen revealed that the tumor was comprised of poorly differentiated adenocarcinoma ((a) H&E ×40) with components of ECL cell carcinoid ((b) H&E ×40). (c) Carcinoid, immunohistochemically positive for chromogranin A staining (arrows), diffusely intermingled with adenocarcinoma (chromogranin A ×4). (d) Both carcinoid and adenocarcinoma invaded to subserosa (H&E ×4).

A 61-year-old man was asymptomatic except for severe anemia (Hb: 4.5 g/dl) that was detected at a medical checkup. Gastroscopy revealed a large gastric tumor near the esophagogastric junction (Figure 6(a)), and biopsy showed poorly differentiated adenocarcinoma with ECL cell tumor (carcinoid). The level of serum gastrin was high (570 pg/mL) and the anti-parietal cell antibody test was negative. After blood transfusion, total gastrectomy was performed (Figure 6(b)) with regional lymphadenectomy. Histological examination of the resected specimen revealed that the tumor was composed of poorly differentiated adenocarcinoma (Figure 7(a)) with components of ECL cell tumors (Figure 7(b)). These ECL cell tumors were diagnosed as carcinoid because they were immunohistochemically positive for chromogranin A (Figure 7(c)) and synaptophysin, and the cell size was larger than small cell carcinoma. The carcinoid diffusely intermingled with adenocarcinoma and invaded into the subserosa (Figure 7(d)). Only adenocarcinoma cells were detected in the metastatic regional lymph nodes. The gastric mucosa was diagnosed as compatible with type A atrophic gastritis.

## 3. Discussion

A gastric carcinoid is a relatively rare neoplasm. Previously, carcinoids have been classified according to the embryologic origin (hindgut, midgut, and foregut); a gastric carcinoid originates from the foregut. Recently, carcinoids have been categorized according to their pathobiological behaviors into 3 different types [2]. Type 1 carcinoid is the most common type; it arises in patients with chronic atrophic gastritis (type A gastritis) under hypergastrinemic conditions. Type 2 carcinoid is the least common type; it occurs in patients with gastrinoma Zollinger-Ellison syndrome (ZES) who have also been diagnosed with multiple endocrine neoplasia syndrome type-I (MEN-I). Type 3 carcinoid occurs sporadically and is not associated with atrophic gastritis or hypergastrinemia; the tumor is usually a single and large tumor and has the most malignant potential among these 3 types of carcinoids. Herein, we report 3 cases of gastric carcinoids. In case 1, the tumor was 2 cm in diameter and invaded the muscularis propria and caused lymph node metastasis. In case 2, the tumors were multiple and small, and in case 3, a carcinoid and adenocarcinoma intermingled in a single tumor that invaded the subserosa. All cases were concomitant with hypergastrinemia and atrophic gastritis. We categorized cases 1 and 2 as type I carcinoid and case 3 as a carcinoid-carcinoma tumor.

A type 1 gastric carcinoid is usually small, multifocal, and located in the gastric fundus with a low potential of malignancy [3]. It grows slowly, and the prognosis is similar to that of the general population. The 5-year survival rate is around 95% [4, 5]. Due to its benign course, the recommended treatment ranges from medical therapy and endoscopic resection with strict follow-up to surgical excision. Gilligan et al. proposed a decision tree for the management of gastric carcinoids [6]. More recently, Borch et al. made recommendations for the treatment of type 1 carcinoids: radical endoscopic removal should be performed for patients with up to 5 tumors with a maximum diameter of 10 mm, and antrectomy should be performed for patients with more than 5 tumors with a maximum diameter of 10 mm [4] (referred in the ENETS guidelines [7]). If the tumors are larger than 10 mm (regardless of number), antrectomy was recommended with surgical excision of tumors larger than 10 mm. If the serosa is involved or the tumor has spread outside the stomach, total gastrectomy with lymph node dissection was advised. Hou et al. suggested that total gastrectomy might be necessary if tumors recurred after antrectomy [8]. 

There seems to be some differences in the characteristics of type 1 gastric carcinoids based on Japanese and Western reports. The incidence of gastric carcinoids is 2.4%–8.7% [9] among all cases of gastrointestinal tract carcinoids in Western countries whereas it is 27.3% in Japan [10]. The prevalence of type 1 gastric carcinoids in Western reports has been around 80% [4, 5, 11]. However, Iwashita reported that type 1 gastric carcinoids accounted only for 35% (25 out of 72 cases) of all cases of gastric carcinoids in Japan [12]. In these 25 cases, all lesions were within the mucosa or submucosa. The rate of lymph node metastasis was 25% and was much higher than that reported in the Western literature (0%–5.9%) [4, 5, 11]. Therefore, Iwashita et al. concluded that the malignant potential of type 1 carcinoids was not as low as it was believed. According to the Western reports, the rate of tumor invasion into the muscularis propria was 3.4%–9.0% and that into the subserosa was 0%–5.9% [4, 5, 11]. In our study, case 1 showed invasion into the muscularis propria with lymph node metastasis, and case 3 showed infiltration of the subserosa. However, both findings were relatively rare. 

In Japan, total gastrectomy with lymph node dissection has been the preferred surgical procedure for multiple gastric carcinoids associated with type A gastritis. The merit of this procedure is that the fundic gland area, which could be the origin of the carcinoid and ECM, is completely resected along with excision of the majority of G cells. Okada et al. also suggested that total gastrectomy should be considered as one of the treatments for multiple gastric carcinoids with hypergastrinemia [13]. In case 2, the patient was recommended to undergo total gastrectomy to remove all tumors. However, we chose antrectomy since it was a typical type 1 gastric carcinoid. ECL cell hyperplasia may be reversible if hypergastrinemia is abolished. Therefore, antrectomy or surgical excision of the majority of the G cells can be an appropriate treatment [14]. The level of serum gastrin actually decreased within normal range after antrectomy in case 2. Some researchers observed the spontaneous regression of residual carcinoids after antrectomy [4]. However, not all patients experienced tumor regression [15]. One reason for that observation was speculated to be an autocrine effect that is present in ECL cells after malignant transformation [16]. Therefore, strict follow-up should be scheduled.

Some authors have recommended a conservative approach without removal of the tumors. Hosokawa et al. proposed a follow-up program without surgical resection for multiple gastric carcinoids that are associated with type A gastritis [17]. They reported 8 patients who were free of growth or metastasis for a maximum of 10.8 years despite persistent hypergastrinemia. They suggested that such patients could be followed up with periodic endoscopic examination if their tumors are smaller than 2 cm in diameter.

In the present study, we observed atrophic gastritis in all 3 cases. The development of adenocarcinoma or endocrine tumors in atrophic gastritis is widely documented [18]. In case 3, the ECL cell component was focally mixed with poorly differentiated adenocarcinoma in one tumor. It may be controversial to diagnose these ECL cell components as carcinoid; however, they were immunohistochemically positive for chromogranin A and synaptophysin, and the cell size was too large to be diagnosed as small cell carcinoma. There have been some reports of carcinoma with interspersed carcinoid, and such a tumor is sometimes referred to as a composite tumor and its occurrence is relatively rare [18]. This phenomenon supports the concept that adenocarcinoma (epithelial) and carcinoid (neuroendocrine) cells can be differentiated from a multipotent multidirectional stem cell. We speculate that high serum gastrin level contributed to this neuroendocrine differentiation. Longstanding hypergastrinemia may have played a causative role in the development of carcinoid and cancer [19]. From this viewpoint, long-acting somatostatin analogs which suppressed gastrin production and reported effective in type 1 carcinoid [20], can be used to slow the malignant change. Another possibility of carcinoid-adenocarcinoma coexistence is that carcinoid releases some growth factors which stimulate gastric mucosa and cause malignant transformation [21]. Moreover, during chronic inflammation at the gastric mucosa, secreted factors such as lymphokines and prostaglandins modulate gastric epithelia, resulting in carcinoid and adenoartinoma [22]. 

We showed 2 cases of multiple gastric carcinoids and 1 case of a carcinoid-adenocarcinoma tumor. All 3 cases were diagnosed with atrophic gastritis, which is associated with an increased risk of gastric carcinoma. We could have selected antrectomy with wedge resection of the large tumor for case 1 and we chose it for case 2. As shown in the previous studies, larger (>10 mm in diameter) or deeper infiltrating (than submucosa) tumors may, although rarely, be associated with regional lymph node metastasis, [10, 12] a prognostic factor of type 1 carcinoid [4]. With intensive follow-up on the small residual carcinoids and the stomach after surgery, we believe that antrectomy (with lymph node dissection in the risky case such as case 1) is a good treatment option regarding the postoperative quality of life.
